# Exposure to Bacterial Signals Does Not Alter Pea Aphids’ Survival upon a Second Challenge or Investment in Production of Winged Offspring

**DOI:** 10.1371/journal.pone.0073600

**Published:** 2013-08-29

**Authors:** Bas ter Braak, Alice M. Laughton, Boran Altincicek, Benjamin J. Parker, Nicole M. Gerardo

**Affiliations:** 1 Department of Biology, Emory University, Atlanta, Georgia, United States of America; 2 Division of Toxicology, Leiden University, Leiden, Netherlands; 3 School of Biological and Chemical Sciences, Queen Mary University of London, London, United Kingdom; 4 Molecular Phytomedicine, University of Bonn, Bonn, Germany; Ecole Normale Supérieur de Lyon, France

## Abstract

Pea aphids have an obligate nutritional symbiosis with the bacteria *Buchnera* *aphidicola* and frequently also harbor one or more facultative symbionts. Aphids are also susceptible to bacterial pathogen infections, and it has been suggested that aphids have a limited immune response towards such pathogen infections compared to other, more well-studied insects. However, aphids do possess at least some of the genes known to be involved in bacterial immune responses in other insects, and immune-competent hemocytes. One possibility is that immune priming with microbial elicitors could stimulate immune protection against subsequent bacterial infections, as has been observed in several other insect systems. To address this hypothesis we challenged aphids with bacterial immune elicitors twenty-four hours prior to live bacterial pathogen infections and then compared their survival rates to aphids that were not pre-exposed to bacterial signals. Using two aphid genotypes, we found no evidence for immune protection conferred by immune priming during infections with either *Serratia marcescens* or with *Escherichia coli*. Immune priming was not altered by the presence of facultative, beneficial symbionts in the aphids. In the absence of inducible immune protection, aphids may allocate energy towards other defense traits, including production of offspring with wings that could escape deteriorating conditions. To test this, we monitored the ratio of winged to unwinged offspring produced by adult mothers of a single clone that had been exposed to bacterial immune elicitors, to live *E. coli* infections or to no challenge. We found no correlation between immune challenge and winged offspring production, suggesting that this mechanism of defense, which functions upon exposure to fungal pathogens, is not central to aphid responses to bacterial infections.

## Introduction

Vertebrates have both adaptive and innate immune systems. The adaptive immune system allows vertebrates to mount highly specialized responses towards microorganisms to which they have been previously exposed. Invertebrates do not have a homolog to vertebrate adaptive immunity. They do however exhibit innate immune responses that are regulated through several immune pathways and through hemocytes, immune cells that are capable of phagocytosing and encapsulating foreign invaders (reviewed in 1,2). Based on the presence of innate but not adaptive immune systems in invertebrates, it was thought that insects would lack the ability to prime the immune system for later invasion by a pathogen to which they had been previously exposed. However, many studies have shown that some insects can have a stronger and more effective immune response after previous pathogen exposure. This phenomenon, known as immune priming, has been demonstrated in response to bacterial, viral and fungal infections [3–7]. In some systems [4,8,9], such immune priming can be transgenerational, meaning that offspring have increased protection against pathogens to which their parents were exposed. The mechanisms underlying insect immune priming remain elusive, though there is evidence for phagocytosis [10–12] and known immune pathways [13,14] being involved.

Recent studies have suggested that pea aphids (*Acyrthosiphon pisum*) have an altered or reduced innate immune repertoire compared to insects such as flies, mosquitoes and bees [15–17]. For example, while pea aphids have some intact pathways known to function in insect immune responses (e.g., Toll and JAK/STAT pathways), they are missing essential peptidoglycan recognition proteins and many genes of the IMD pathway [16]. Genome-wide searches, proteomic assays and traditional bacterial clearance assays have yet to identify functional antimicrobial peptides [16], which form the basis of response to bacterial pathogens in many insects (though see 18). Pea aphids also exhibit weak lysozyme activity, hemolymph coagulation, and phenoloxidase activation [17,19]. Pea aphids, however, do possess several putative immune cell types, some of which can phagocytose bacteria [19,20].

Though aphid immune responses appear limited, another possibility is that they possess alternative immune mechanisms, some of which could require priming for activation. To begin to assess the potential for priming-mediated protection, we exposed aphids to a mixture of Gram-positive and Gram-negative bacterial elicitors (i.e., heat-killed bacteria that provide the infection signal without pathogenesis) and then compared the survival of these aphids upon live pathogen infection to the survival of aphids that were not pre-exposed to such elicitors. To assess whether immune priming would be altered by the presence of beneficial bacterial symbionts, which are common in pea aphids [21] and that potentially could be harmed by any upregulated responses towards bacterial pathogens, we utilized both aphids both harboring and not harboring facultative (secondary) symbionts. Finally, we addressed the possibility that exposure could elicit non-immunological forms of defense, such as the production of winged offspring. Winged offspring production is increased in response to the presence of predators [22] and to fungal pathogen infection [23], and was therefore hypothesized to be a potential defense mechanism employed against bacterial infection, enabling offspring to fly to areas with lower pathogen prevalence.

## Materials and Methods

### Aphid Lines

*Acyrthosiphon pisum* (Hemiptera, Aphididae) clones were maintained on 2-3 week old fava bean plants in 16 h light: 8 h dark conditions at 20°C. We transferred 5-10 aphids to new plants on a weekly basis. We used aphid genotypes 5A0, originally collected in 1999 in Madison, Wisconsin, and LSR1, originally collected near Ithaca, New York in 1998. Both of these contain the primary obligate bacterial symbiont, *Buchnera* *aphidicola* , but no facultative symbionts. To test for interactions between facultative symbionts, immune priming, and pathogen infection, we also used aphid lines of the 5A genetic background that have facultative symbionts (5AR = *Serratia* *symbiotica* , 5AT = *Hamiltonelladefensa* , 5AU = *Regiellainsecticola* ) [24].

### Pre-exposure to Pathogen Signals

For pre-exposure prior to live infection, bacterial elicitors were obtained by autoclaving (20 min at 121°C and 1.5 bar pressure) a mixture of approximately 10^8^ *E. coli* cells and 10^8^ *Micrococcus luteus* cells in 1 mL of phosphate buffered saline (PBS) prior to use. A mixture of both bacteria ensured the presence of a broad array of elicitors, including pattern molecules from both Gram-negative and Gram-positive bacteria. Bacterial inoculums were injected dorsolaterally through the abdominal wall into the hemocoel of young adult (10-12 day old), asexual, unwinged aphid females using minutin insect needles dipped into the bacterial solution. Control (not primed) aphids were stabbed with a sterile needle dipped in PBS.

### *Serratia Marcescens* Infections

In a set of three experiments (Experiments 1-3, Table 1), twenty-four hours after the pre-exposure treatment, we infected aphids with live *Serratia* cf. *marcescens* strain RHOD, which was originally isolated from a naturally infected aphid (samples sizes listed in Table 1). Though not an aphid-specific pathogen, *S. marcescens* has been isolated from laboratory-reared aphids (Gerardo, unpublished data) and is known to cause high mortality [25]. Bacteria grown in Luria broth (LB) to an optical density (OD) of 1 at 600 nm were centrifuged and bacterial pellets were washed with PBS and resuspended in 1/10^th^ volume of PBS, yielding a dense bacterial solution. Bacterial inoculums were injected dorsolaterally through the abdominal wall into the hemocoel using minutin insect needles dipped into bacterial solutions. In general, this procedure results in inoculums of approximately 50 to 300 bacteria per aphid (data not shown) when the needle penetrates in approximately 0.5 mm and parallel to the cuticle. After stabbing, aphids were placed in sterile Petri dishes for 30 minutes and then transferred to fava bean plants and monitored for survival. In Experiment 1, we used two aphid genotypes (5A0, LSR1) without facultative symbionts. Half of the individuals were stabbed with either live *S. marcescens* or, for control treatments, with heat-killed *S. marcescens* (autoclaved 20 min at 121°C and 1.5 bar pressure) in PBS. To confirm our results, in a second, monofactorial experiment (Experiment 2), using a single aphid line (5AR), with the facultative symbiont *Serratia* *symbiotica* , all aphids were infected with live *S. marcescens* (no aphids received a heat-killed bacterial treatment as the second challenge). Similarly, in Experiment 3, using a different aphid line (LSR1), all aphids were infected with live *S. marcescens* (no aphids received a heat-killed bacterial treatment as the second challenge).

**Table 1:** Samples sizes for infection experiments.

**Experiment**	**Line**	**Facultative Symbiont**	**Pre-exposure**	**Live Challenge**	**n**
Experiment 1	5A0	none	bacterial elicitors	*S. marcescens*	13
	5A0	none	bacterial elicitors	control	14
	5A0	none	control	*S. marcescens*	14
	5A0	none	control	control	13
	LSR1	none	bacterial elicitors	*S. marcescens*	14
	LSR1	none	bacterial elicitors	control	14
	LSR1	none	control	*S. marcescens*	11
	LSR1	none	control	control	12
Experiment 2	5AR	*S* *. symbiotica*	bacterial elicitors	*S. marcescens*	35
	5AR	*S* *. symbiotica*	control	*S. marcescens*	35
Experiment 3	LSR1	none	bacterial elicitors	*S. marcescens*	36
	LSR1	none	control	*S. marcescens*	34
Experiment 4	5A0	none	bacterial elicitors	*E. coli*	4
	5A0	none	bacterial elicitors	control	5
	5A0	none	control	*E. coli*	4
	5A0	none	control	control	6
	5AR	*S* *. symbiotica*	bacterial elicitors	*E. coli*	5
	5AR	*S* *. symbiotica*	bacterial elicitors	control	6
	5AR	*S* *. symbiotica*	control	*E. coli*	5
	5AR	*S* *. symbiotica*	control	control	7
	5AT	*H* *. defensa*	bacterial elicitors	*E. coli*	5
	5AT	*H* *. defensa*	bacterial elicitors	control	5
	5AT	*H* *. defensa*	control	*E. coli*	9
	5AT	*H* *. defensa*	control	control	6
	5AU	*R* *. insecticola*	bacterial elicitors	*E. coli*	5
	5AU	*R* *. insecticola*	bacterial elicitors	control	7
	5AU	*R* *. insecticola*	control	*E. coli*	4
	5AU	*R* *. insecticola*	control	control	5
Experiment 5	5AR	*S* *. symbiotica*	bacterial elicitors	*E. coli*	35
	5AR	*S* *. symbiotica*	control	*E. coli*	35
Experiment 6	LSR1	none	bacterial elicitors	*E. coli*	35
	LSR1	none	control	*E. coli*	36

### *Escherichia Coli* Infections

In a set of three experiments (Experiments 4-6, Table 1), twenty-four hours after the pre-exposure treatment, we infected aphids with live *Escherichia coli* K-12 strain MG1655 [26] (samples sizes listed in Table 1). Though also not an aphid-specific pathogen, *E. coli* has been isolated from laboratory-reared aphids (Gerardo, unpublished data) and is known to cause high mortality [27]. Bacterial preparation and inoculations were conducted as above. In Experiment 4, we infected aphids of a single genotype, both with and without facultative symbionts (line 5A0, 5AR, 5AT, 5AU), with either live *E. coli* or inoculated them with heat-killed *E. coli*. In Experiment 5, using a single aphid line (5AR), all aphids were infected with live *E. coli* (no aphids received a heat-killed bacterial treatment as a second challenge). In Experiment 6, using a different aphid line (LSR1), all aphids were infected with live *E. coli* (no aphids received a heat-killed bacterial treatment as a second challenge)*.*

### Production of Winged versus Unwinged Offspring

To determine whether exposure to bacterial elicitors or to live bacterial infections could stimulate exposed mothers to produce a greater proportion of winged offspring, 40 12-day old, unwinged, reproductive, asexual LSR1 aphids were split across four treatments: 1) no stab control, 2) sterile stab (PBS) control, 3) stab with heat-killed mix of Gram-positive and Gram-negative bacteria, 4) stab with live *E. coli*. Heat-killed bacteria elicitors were prepared as above. To increase the time to death for aphids infected with live bacteria, *E. coli* was grown to an OD_600_ of 0.5 in LB and then resuspended in PBS. After stabbing, aphids were placed in sterile Petri dishes for 30 minutes and then transferred to individual fava bean plants. Every 24 hours, for nine days, we counted the number of offspring produced. Adult aphids were transferred to new plants every three days. Their offspring were scored as winged or unwinged when they were fourth instars (when wing buds can be seen with the naked eye) or young adults (when wings are fully formed).

### Statistical Analyses

Survival analyses using Cox proportional hazards models (with censoring) were carried out using the coxph function of the Survival package of R (version 2.13), following assessment of proportional hazards using cox. zph [28,29]. For Experiment 1, cofactors included aphid genotype, pre-exposure and infection treatment upon second challenge. For Experiment 4, cofactors included symbiont type, pre-exposure and infection treatment upon second challenge. For the monofactorial experiments (Experiments 2-3, 5-6), the only factor was pre-exposure. Minimal models were derived by removing model terms and then conducting model comparisons using likelihood ratio tests. Terms were retained in the minimal model if their removal significantly reduced the explanatory power of the model [30]. For Experiments 1 and 4, one of the factors, infection treatment, did not meet the proportional hazards assumption, and so we used a stratified Cox procedure to analyze these data sets.

Statistical analysis of whether exposure impacted the proportion of offspring that were winged was carried out in R using a general linear model with a quasibinomial distribution. Data on the number of winged and unwinged offspring produced by control, sterile-stabbed and heat-killed bacteria-stabbed mothers that did not survive the entirety of the experiment were excluded from analyses; included were numbers of winged and unwinged offspring for eight full control (no stab), eight sterile stab and nine heat-killed bacteria treated aphids. Offspring data from four of the ten aphids presumably stabbed with live *E. coli* that did not die over the course of the experiment were also excluded as we cannot guarantee that these mothers were successfully infected; these four mothers only produced one winged offspring out of 252 total offspring. To determine if the proportion of winged offspring was significantly impacted only in one window of reproduction, we repeated the analysis separately for offspring produced 1 to 3 days after treatment, 4 to 6 days after treatment, and 7 to 9 days after treatment, using either a binomial or quasibinomial distribution depending of the degree of overdispersion. The 7 to 9 day analysis excluded the live bacteria treatment, because all aphids presumed to be successfully infected with live bacteria had died by this time point.

## Results

### Immune Priming and *Serratia Marcescens* Infections

**Figure 1:**
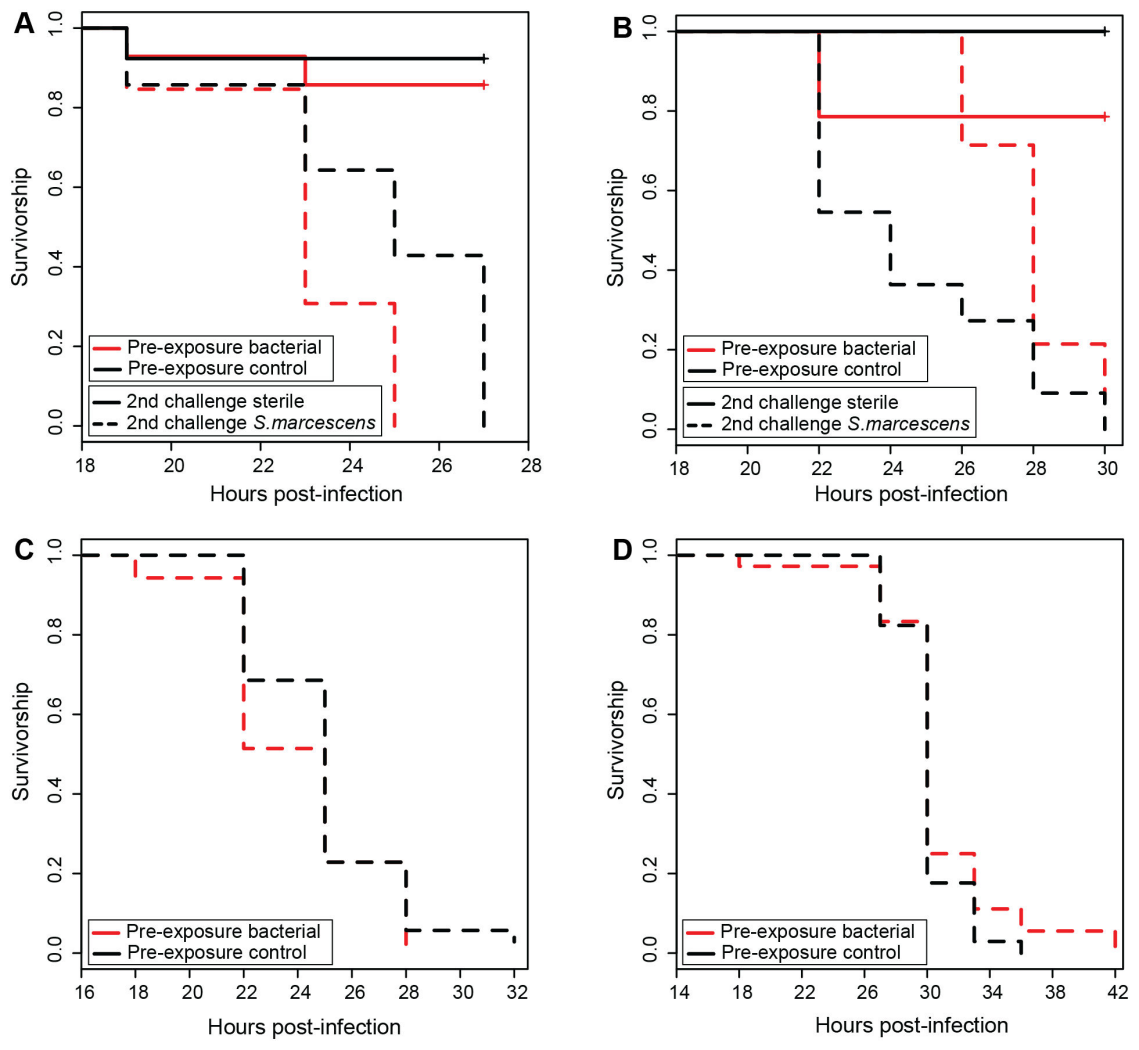
Aphid survival in relation to pre-exposure with bacterial elicitors and subsequent challenge with *S. marcescens*. A) B) In Experiment 1, while *S. marcescens* reduced aphid survival (pathogen-infected treatments are dotted, uninfected treatments are solid), the interaction between pre-exposure to bacterial elicitors (pre-exposed treatments are red, unprimed treatments are black) and subsequent challenge with live *S. marcescens* was not significant. A) Experiment 1, aphid genotype 5A0. B) Experiment 1, aphid genotype LSR1. C) In follow-up Experiment 2, giving all aphids (line 5AR) a subsequent challenge with live *S. marcescens*, pre-exposure did not impact survival. D) In follow-up Experiment 3, giving all aphids (genotype LSR1) a subsequent challenge with live *S. marcescens*, pre-exposure did not impact survival.

For Experiment 1 (Figure 1A, B), the minimal model indicated a marginally non-significant interaction between aphid genotype, pre-exposure, and infection treatment upon second challenge (p = 0.05), which is reflected by the somewhat different responses of the two aphid genotypes (5A0, LSR1) to pre-exposure and subsequent live bacterial challenge (Figure 1A,B). Upon second challenge with live bacteria, 5A0 aphids appear to have similar survival curves regardless of pre-exposure treatment (Figure 1A), whereas LSR1 aphids had slightly increased survival if they were pre-exposed to bacterial signals (Figure 1B). There was also a significant two-way interaction between genotype and pre-exposure treatment (p = 0.01), which may reflect the considerable natural variation in aphid responses to challenge, as has been shown previously [27]. Of importance here, regardless of whether the three-way interaction was retained in the model, the two way interaction between pre-exposure treatment and infection treatment upon second challenge was not significant (p > 0.09), which indicates that pre-exposure did not provide protection upon subsequent challenge with live bacteria. Experiment 2 (Figure 1C) and Experiment 3 (Figure 1D), with larger samples sizes and simpler, monofactorial designs, indicated no impact of pre-exposure on survival after subsequent live bacterial infections for either 5AR (Experiment 2, p = 0.28) or for LSR1 (Experiment 3, p = 0.27) aphids.

### Immune Priming and *Escherichia Coli* Infections

**Figure 2:**
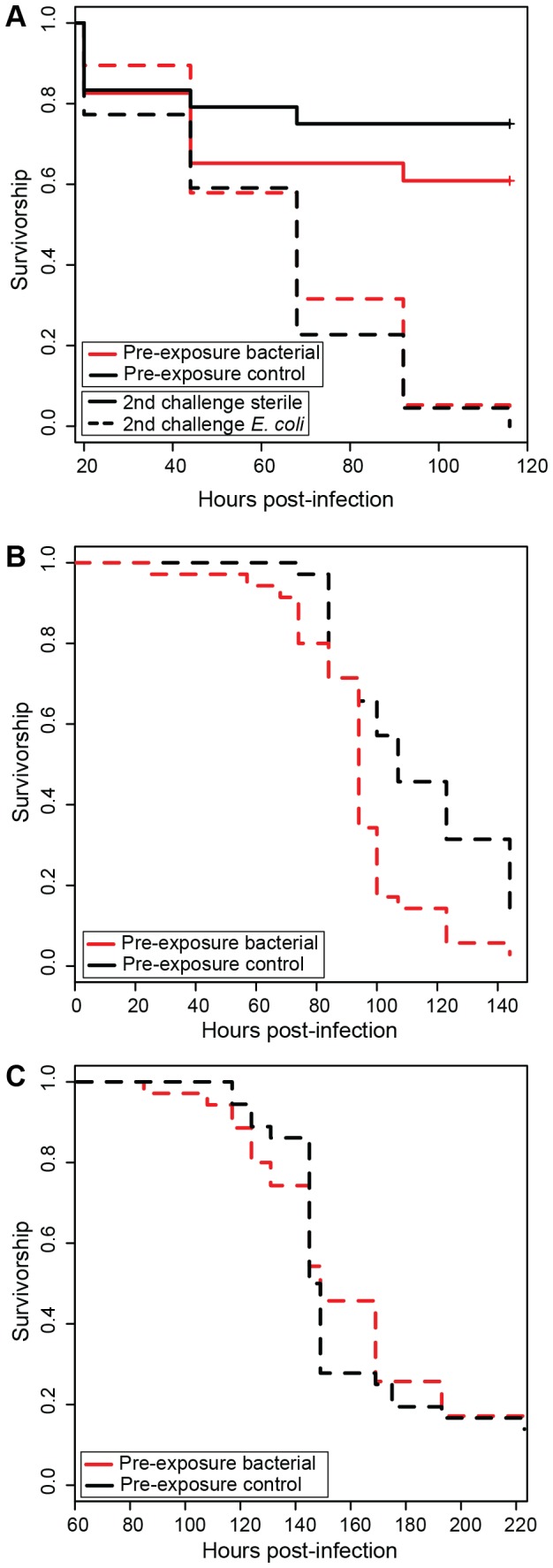
Aphid survival in relation to pre-exposure with bacterial elicitors and subsequent challenge with *E. coli*. A) In Experiment 4, while *E. coli* reduced aphid survival (pathogen-infected treatments are dotted, uninfected treatments are solid), there was no significant interaction between challenge with live bacteria and pre-exposure to bacterial elicitors (pre-exposed treatments are red, unprimed treatments are black). As harboring facultative symbionts did not significantly alter survival, aphids with and without facultative symbionts are pooled. B) In follow-up Experiment 5, giving all aphids (line 5AR) a subsequent challenge with *E. coli*, pre-exposure with bacterial elicitors decreased survival. C) In follow-up Experiment 6, giving all aphids (genotype LSR1) a subsequent challenge with *E. coli*, pre-exposure did not impact survival.

For Experiment 4 (Figure 2A), the minimal model indicated no significant interactions between cofactors, and, in particular, the interaction between pre-exposure and challenge was not significant (p = 0.27), which would be expected if pre-exposure increased survival upon a second challenge with live bacteria. In Experiment 5 (Figure 2B), using aphid clone 5AR, pre-exposure had a significant impact on survival after subsequent challenge (p < 0.01), but not in a manner consistent with immune priming based protection; aphids pre-exposed to bacterial signals had lower survival than those not pre-exposed to bacterial signals. In Experiment 6 (Figure 2C), using aphid genotype LSR1, pre-exposure had no significant impact on survival after a second challenge with live *E. coli* (p = 0.78).

### Production of Winged versus Unwinged Offspring in Response to Pathogen Exposure

**Figure 3:**
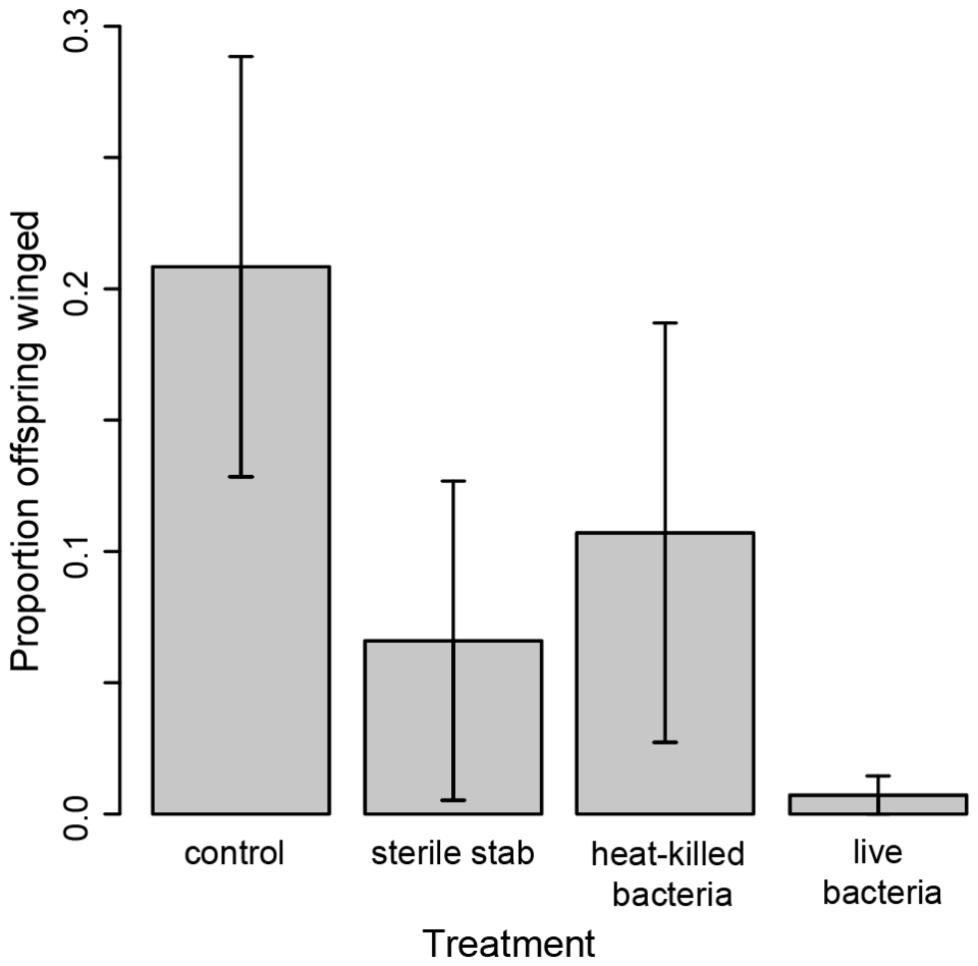
Impact of challenge on offspring production. Mothers exposed to bacteria and to bacteria elicitors did not produce more winged offspring than unexposed mothers. Young adult females were not stabbed (control) or were stabbed with needles dipped in PBS (sterile stab), a heat-killed bacterial solution or a solution of live *E. coli* (live bacteria). Includes all offspring produced within 9 days of challenge. Error bars = s.e.m.

We found that aphids of genotype LSR1, which readily produce winged aphids upon exposure to aphid alarm pheromone and crowing conditions (data not shown), did not increase production of winged offspring in response to exposure to either bacterial signals or to live *E. coli* pathogen infection. While 75% of control aphids had at least one winged offspring, only 37% of sterile stabbed aphids, 33% of aphids stabbed with heat killed bacteria, and 16% of aphids stabbed with live bacteria produced any winged offspring. Statistical analysis of the average proportion of winged versus unwinged offspring across all 9 days indicated no statistical differences between treatments (Figure 3, F_3,30_ = 1.12, p = 0.36). We also found no effect of treatment within any of the shorter windows of reproduction (days 1 to 3: F_3,28_ = 1.47, p = 0.24; days 4 to 6: F_3,28_ = 0.81, p = 0.50; days 7 to 9: F_3,24_ = 0.63, p = 0.54).

## Discussion

Immune priming decreases susceptibility of some arthropods to some pathogens [3–5,10,31,32], but it is not universal to all insect-pathogen interactions [33,34]. Here, we saw no evidence of pre-exposure to bacterial elicitors impacting survival against two bacterial pathogens, even when, in the case of *E. coli* infection, elicitors from that specific bacterial strain were present in the priming solution. We of course cannot assume that immune priming does not occur against other microbial challenges in aphids, particularly since in other systems (e.g., Tribolium beetles [32]) there is evidence for insect immune priming against some microbes and not others. However, we can conclude that priming does not appear to be a component of aphid immune responses towards bacteria in general.

While we did not test transgenerational immune priming in the traditional sense (i.e., we did not test whether the offspring of pre-exposed mothers had increased survival upon bacterial exposure), we did test whether increased production of winged offspring, a potential non-immunological transgenerational defense, could be part of the aphid response to these bacteria. Upon exposure to signals of danger (e.g., alarm pheromones released in the presence of predators, fungal pathogens) and deteriorating conditions (e.g., crowding on the host plant), aphids increase the proportion of winged offspring that they produce [22,23,35,36]. We found no evidence that LSR1 aphids, a clone that readily produces winged offspring in response to alarm pheromone, produce more winged offspring in response to either a mixture of Gram-positive and Gram-negative bacterial elicitors, or to infection with live *E. coli*. While this does not preclude the possibility of such a response in other aphid genotypes or in response to other aphid bacterial pathogens, it does suggest that winged offspring production is not a general response to bacterial invasion. Previous work has shown that at least some aphids, including LSR1, increase the rate of reproduction after pathogen exposure, a mechanism of defense known as fecundity compensation [17,37]. Though our experiment was not designed to test for fecundity compensation, our results were consistent with previous findings; namely, we saw a trend for an increased rate of reproduction in response to exposure to bacterial elicitors relative to a sterile stab (data not shown). Fecundity compensation suggests that aphids do have the ability to recognize and respond to bacterial infection.

If immune priming had provided protection to aphids against bacteria in these experiments, it may have helped explain reasons for the limited transcriptional and proteomic immune responses of aphids towards bacterial pathogens seen previously in the absence of priming [16,17]. Given that we found no evidence of immune priming against the two tested bacteria, other approaches will be needed to gain better insight into the immunological capacity of aphids and other hemipterous insects, a group where little is yet known about immunity, but where interactions with both beneficial and harmful microbes fundamentally shape their ecology and evolution.
